# Recurrent staphylococcal scalded skin syndrome in a 20‐month old—A case report

**DOI:** 10.1002/ccr3.7805

**Published:** 2023-08-17

**Authors:** Camille Basurto, Nada Baah‐Owusu, Kyla Berreth

**Affiliations:** ^1^ Liberty College of Osteopathic Medicine Lynchburg Virginia USA; ^2^ Sovah Pediatrics—Danville Danville Virginia USA

**Keywords:** atopic dermatitis, exotoxins, staph scalded skin syndrome, staphylococcus, toxic epidermal necrolysis

## Abstract

We present a case of a 20‐month‐old child with a history of atopic dermatitis that exhibited recurrent erythematous‐bullous lesions consistent with Staphylococcal Scalded Skin syndrome (SSSS). SSSS is an exfoliative toxin‐mediated skin disorder most commonly found in children. In this paper, we discuss the importance of recognizing the clinical symptomatology and progressive nature of SSSS, particularly in patients with a history of atopic dermatitis, to ensure prompt treatment and resolution of the syndrome.

## Graphical abstract

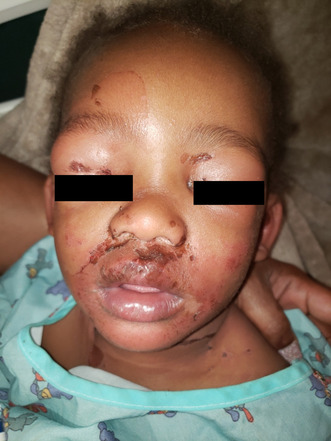

## 1 BACKGROUND

Staphylococcal Scalded Skin Syndrome (SSSS) is an exfoliative skin disease usually due to a toxin produced by a *Staphylococcus aureus* infection.^1^ Toxin a or b from *S. aureus* spread hematogenously and have been shown to lyse the cell–cell adhesion molecule desmoglein‐1 in the superficial epidermis, causing intraepidermal splitting and thus a loss of structural integrity.^2^ SSSS primarily occurs in children and neonates and is rare in adults.^3^, ^4^, ^5^ We present a case of a 20‐month‐old patient who presented with progressive SSSS and recurrence of SSSS to emphasize the role of early treatment and diagnosis in reducing the morbidity of the disease.

## 2 CASE PRESENTATION

### 2.1 Initial admission

**FIGURE 1:**
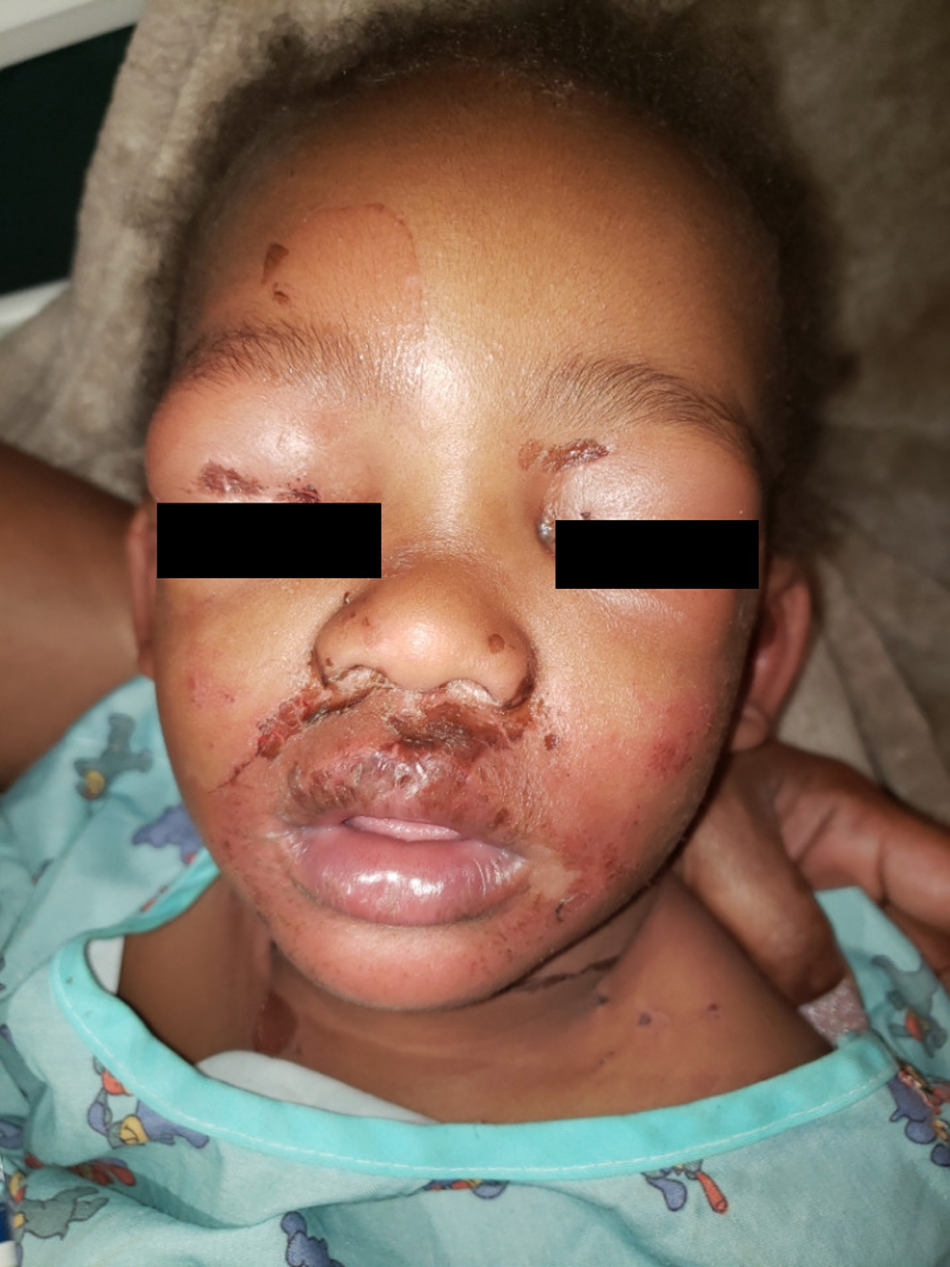
Day 1 of first admission showing facial and bilateral periorbital edema with diffuse erythematous patches with overlying hemorrhagic crust.

Day 1: A 19‐month‐old African American male presented to the emergency department (ED) with erythematous patches and blistering on his face and neck as well as erythematous patches on his buttocks (Figure 1). Patient had a history of atopic dermatitis primarily affecting bilateral antecubital fossa managed with triamcinolone acetonide 0.1% cream as needed for up to 2 weeks. The patient had no new medications prior to this event and was up to date on relevant immunizations.

Per caregivers who were present at ED, the patient was also irritable, had reduced appetite, and appeared fatigued 3–5 days before presentation. The patient also experienced 2 days of afebrile dry cough, rhinorrhea, and sinus congestion, increased scratching of the antecubital fossa due to his atopic dermatitis. Upon rubbing of the perinasal and perioral area in the ED, the uppermost layer of skin sloughed off with minimal pressure (positive Nikolsky's sign). On physical exam, erythema and edema were present. The mucous membranes of the mouth and pharynx were not affected. The patient was apyretic. The patient's initial white blood cell count (WBC) was 8.3 × 10^9/L. We obtained blood and ear mucosal wound cultures for further investigation. A diagnosis of SSSS was made based on history and clinical features. The diagnoses of bullous impetigo and toxic epidermal necrolysis (TEN) were also considered, but the lack of mucous membrane involvement in our patient made SSSS more likely. A skin biopsy was not performed as the clinical presentation was consistent with SSSS.

The patient received IV nafcillin 40 mg/kg/day every 6 h after admission as well as intravenous fluids. For pain control, the patient was given ibuprofen and acetaminophen as needed. The patient's wounds were covered with sterile bandages.

Days 2–3: After discussion with the caregivers, on day 2 the patient was started on IV clindamycin 30 mg/kg/day to target the exfoliative toxin of *S. aureus*. After 48 h, the results of the blood and ear mucosal wound cultures were negative, and the urine analysis was clear. The patient's pain was controlled. However, the patient's caregivers noticed increased periorbital edema without drainage on day 2. The edema was thought to be due to third spacing in the setting of inflammation, and fluids were decreased to keep vein open (KVO).

**FIGURE 2:**
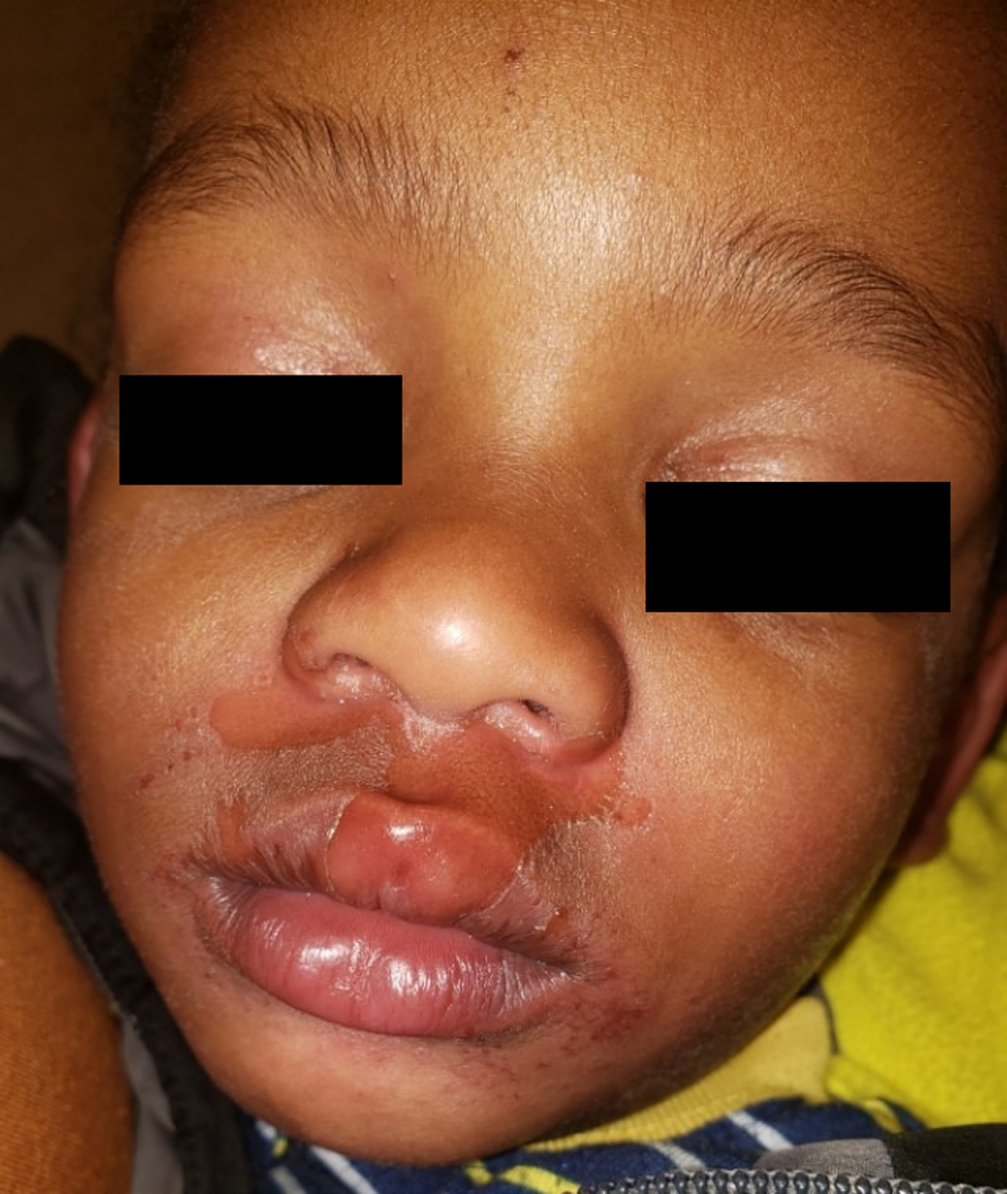
Day 3 of first admission showing resolving facial and bilateral periorbital edema and improving erythematous patches.

Of note, the patient's WBC count of 5.0 × 10^9 /L (ANC 0.9) on hospital day 3 was thought to be caused by IV nafcillin. Repeat complete blood count (CBC) within 24 h showed WBC count of 5.1 (absolute neutrophil count, ANC, 1.2). On day 3, periorbital edema was decreased, and skin lesions were healing with crusting (Figure 2). The patient was taking adequate oral intake, and the IV antibiotics were transitioned to oral cephalexin, and clindamycin was discontinued. The patient was discharged on an appropriate 7‐day course of oral cephalexin, topical mupirocin, and close outpatient pediatric follow‐up.

Readmission, 3 weeks later: Subsequently, the now 20‐month‐old patient presented to the ED with erythematous patches on face, neck, hyperpigmentation on buttocks consistent with SSSS. Patient's caregivers stated that patient's skin was clear for 2 weeks after initial admission and treatment, and the patient continued to scratch antecubital fossa at night due to atopic dermatitis. The patient's mother noticed an erythematous patch behind the ear, which had worsened over the past 2 days, so she brought the patient to the ED. At readmission, there was also no involvement of mucosal membranes and thus no concern for Steven's Johnson Syndrome (SJS). CBC and complete metabolic panel (CMP) were obtained in the ED and were in the normal range except for a low Na, 133 mmol/L. Blood cultures were obtained, showing no growth after 48 h; wound cultures identified no organisms. Initially, the patient was given IV nafcillin for Methicillin‐sensitive *Staphylococcus aureus* (MSSA) coverage because, during the prior admission, this treatment healed the rash.

After discussion with the Pediatric Infectious Disease team, on day 2 the patient was switched from IV nafcillin to a 10 day course of PO Linezolid. The Pediatrics ID team was concerned that, due to the rapid recurrence of SSSS, this patient may be experiencing a rare case of Methicillin‐resistant *Staphylococcus aureus* (MRSA) caused SSSS. Thus, the coverage needed to be broadened.

On day 3, the patient had fewer new lesions, and previous lesions appeared to be healing with scabbing and hyperpigmentation. He was afebrile during his entire hospital stay. The patient was also well appearing with adequate by mouth (PO) intake during the hospital stay and was discharged on day 3 with close outpatient pediatric follow up. The patient's mother was appropriately concerned about immune deficiency, given the recurrence of SSSS. Pediatrics Infectious Disease commented that his readmission was more likely due to MRSA rather than MSSA, which is why SSSS recurred. The patient was also otherwise a normally developing child with excellent growth. The patient's mother knew she needed to return should symptoms worsen or reoccur.

## 3 DISCUSSION

In this case report, the prompt diagnosis and treatment of SSSS during the first ED admission allowed the patient to heal, and the subsequent readmission with broadened antibiotic coverage allowed for timely discharge and effective treatment.

SSSS, or Ritter's disease, is a rare disease process that typically occurs in children. The preceding *S. aureus* infection spreads in the bloodstream and lyses the desmoglein‐1 proteins in the stratum granulosum layer of the epidermis.^1^, ^2^, ^6^ This separation of the anchoring desmosomes leads to the exfoliative presentation of SSSS. SSSS typically arises from an area of infection, such as impetigo, bacterial conjunctivitis, or iatrogenic wounds.^7^ The susceptibility of children, particularly neonates, to the acquisition of SSSS has been postulated to result from the lack of protective antibodies to *S. aureus* toxins and/or the insufficient excretion of exotoxins from children's kidneys.^1^

The diagnosis of SSSS is mainly clinical. Prodromal symptoms of irritability, skin pain, fever, and poor feeding are common. Cutaneous erythema may initially present in the skin folds of the neck, axillae, and gluteal region before becoming generalized within the first 48 hours of presentation.^7^ On exam, the skin may have flaccid bullae that erode upon minimal pressure. Additionally, superficial desquamation occurs due to the exotoxin's lytic mechanism.^6^ There is typically a quick progression of the disease process.

The standard treatment of SSSS includes IV antibiotics against staphylococcal species (e.g., Nafcillin, oxacillin) and supportive care.^6^, ^7^, ^8^, ^9^ Empiric treatment with penicillinase‐resistant penicillins is done initially, with broadening for MRSA coverage considered when clinical improvement is not observed or in communities with high MRSA incidence.^10^ In our case, MRSA coverage (PO linezolid) was added after initial clearing and improvement was followed by a recurrence of SSSS. Clarithromycin can be given to patients with a penicillin allergy.^8^ Other therapies, such as IV immunoglobulin, have been suggested to antagonize the exfoliative toxins of SSSS.^11^ Patients may continue to have skin pain following treatment as well as post‐inflammatory hypopigmentation or hyperpigmentation during the healing process.^6^ SSSS risks progressing to sepsis if not recognized and treated early. Other complications include secondary infection, dehydration, electrolyte imbalance, and death.^5^, ^12^

Recurrent SSSS in the pediatric population is rare, and a literature review revealed recurrent SSSS documented more often in the neonate population than in infants or older pediatric patients. Specifically, five reported cases occurred in neonates in either healthy full‐term, preterm, or low birth‐weight neonates.^13^, ^14^, ^15^, ^16^, ^17^ One of these case reports details two Taiwanese siblings with Netherton syndrome, a condition that impairs skin barrier protection, and immune response.^17^ We found one case report of two children in Tanzania, aged 5 and 10, with recurrent SSSS.^18^ Our 20‐month‐old pediatric patient is a unique case of recurrent SSSS in a toddler‐aged patient.

The differential diagnosis for SSSS includes TEN and SJS.^5^ TEN and SJS have mucous membrane and cutaneous involvement, which was absent here. Bullous impetigo is also on the differential diagnosis, but bullous impetigo has localized skin infection rather than hematogenous spread. The former has more limited involvement and a less severe clinical presentation.^6^

## 4 CONCLUSION

This case report highlights the importance of prompt evaluation and antibiotic treatment as well as the consideration of MRSA in SSSS. More research is necessary to elucidate the prevalence and resistant staphylococcal strains and therapies for SSSS caused by MRSA. This case report aims to support research in exfoliative skin conditions further.

## AUTHOR CONTRIBUTIONS

**Camille Basurto:** Conceptualization; data curation; formal analysis; investigation; methodology; project administration; writing – original draft; writing – review and editing. **Nada Baah‐Owusu:** Investigation; project administration; writing – original draft; writing – review and editing. **Kyla Berreth:** Investigation; project administration; writing – original draft; writing – review and editing.

## FUNDING INFORMATION

None.

## CONFLICT OF INTEREST STATEMENT

The authors have no disclosures to report.

## CONSENT

Patient's guardians provided written consent for the use of patient photographs and related materials for publication.

## Data Availability

None.

## References

[1] Handler MZ , Schwartz RA . Staphylococcal scalded skin syndrome: diagnosis and management in children and adults. J Europ Acad Dermatol Venereol. 2014;28(11):1418‐1423. 10.1111/jdv.12541 24841497

[2] Amagai M , Matsuyoshi N , Wang ZH , Andl C , Stanley JR . Toxin in bullous impetigo and staphylococcal scalded‐skin syndrome targets desmoglein 1. Nat Med. 2000;6(11):1275‐1277. 11062541 10.1038/81385

[3] Ladhani S , Evans RW . Staphylococcal scalded skin syndrome. Arch Dis Child. 1998;78(1):85‐88. 9534685 10.1136/adc.78.1.85 PMC1717447

[4] Staiman A , Hsu DY , Silverberg JI . Epidemiology of staphylococcal scalded skin syndrome in US adults. J Am Acad Dermatol. 2018;79(4):774‐776. 29902545 10.1016/j.jaad.2018.06.008

[5] Staiman A , Hsu DY , Silverberg JI . Epidemiology of staphylococcal scalded skin syndrome in US children. Br J Dermatol. 2018;178(3):704‐708. 29077993 10.1111/bjd.16097

[6] Lina G , Gillet Y , Vandenesch F , Jones ME , Floret D , Etienne J . Toxin involvement in staphylococcal scalded skin syndrome. Clin Infec Dis. 1997;25(6):1369‐1373. 9431380 10.1086/516129

[7] Mcmahon P . In: Ofori A , ed. Staphylococcal scalded skin syndrome. UptoDate; 2022 Available from https://www.uptodate.com/contents/staphylococcal‐scalded‐skin‐syndrome

[8] Leung A , Barankin B , Leong KF . Staphylococcal‐scalded skin syndrome: evaluation, diagnosis, and management. World J Pedia. 2018;14(2):116‐120. 10.1007/s12519-018-0150-x 29508362

[9] Li MY , Hua Y , Wei GH , Qiu L . Staphylococcal scalded skin syndrome in neonates: an 8‐year retrospective study in a single institution. Pediatr Dermatol. 2014;31(1):43‐47. 23557104 10.1111/pde.12114

[10] Wang Z , Feig JL , Mannschreck DB , Cohen BA . Antibiotic sensitivity and clinical outcomes in staphylococcal scalded skin syndrome. Pediatr Dermatol. 2020;37(1):222‐223. 31626359 10.1111/pde.14014

[11] Takei S , Arora YK , Walker SM . Intravenous immunoglobulin contains specific antibodies inhibitory to activation of T cells by staphylococcal toxin superantigens [see comment]. J Clin Invest. 1993;91(2):602‐607. 8432865 10.1172/JCI116240 PMC287991

[12] Patel GK . Treatment of staphylococcal scalded skin syndrome. Expert Rev Anti Infect Ther. 2004;2(4):575‐587. 15482221 10.1586/14787210.2.4.575

[13] Davidson J , Polly S , Hayes PJ , Fisher KR , Talati AJ , Patel T . Recurrent staphylococcal scalded skin syndrome in an extremely low‐birth‐weight neonate. AJP Reports. 2017;7(2):e134‐e137. 28674637 10.1055/s-0037-1603971 PMC5493488

[14] Bhavsar I , Hayes R , Vaughan A . Case report: recurrent staphylococcal scalded skin syndrome in healthy term neonate despite full course of antibiotic therapy. Marshall Journal of Medicine. 2016;2(1):25.

[15] Duijsters CE , Halbertsma FJ , Kornelisse RF , Arents NL , Andriessen P . Recurring staphylococcal scalded skin syndrome in a very low birth weight infant: a case report. J Med Case Reports. 2009;3(1):1‐3. 10.4076/1752-1947-3-7313 PMC2737800 19830179

[16] Rieger‐Fackeldey E , Plano LR , Kramer A , Schulze A . Staphylococcal scalded skin syndrome related to an exfoliative toxin A‐and B‐producing strain in preterm infants. Eur J Pediatr. 2002;161:649‐652. 12447663 10.1007/s00431-002-1080-z

[17] Chao SC , Richard G , Lee JY . Netherton syndrome: report of two Taiwanese siblings with staphylococcal scalded skin syndrome and mutation of SPINK 5. British J Dermatol. 2005;152(1):159‐165. 10.1111/j.1365-2133.2005.06337.x 15656819

[18] Machang'u RS , Mgode G , Gisakanyi N . Recurrent staphylococcal scalded skin syndrome in children: report of two cases. East Afr Med J. 1997;74(9):603‐604. 9487441

